# Returning long-range PM_2.5_ transport into the leeward of East Asia in 2021 after Chinese economic recovery from the COVID-19 pandemic

**DOI:** 10.1038/s41598-022-09388-2

**Published:** 2022-04-01

**Authors:** Syuichi Itahashi, Yuki Yamamura, Zhe Wang, Itsushi Uno

**Affiliations:** 1 Sustainable System Research Laboratory (SSRL), Central Research Institute of Electric Power Industry (CRIEPI), Abiko, Chiba 270-1194 Japan; 2 Fukuoka Institute of Health and Environmental Science, Dazaifu, Fukuoka 818-0135 Japan; 3 State Key Laboratory of Atmospheric Boundary Layer Physics and Atmospheric Chemistry (LAPC), Institute of Atmospheric Physics (IAP), Chinese Academy of Sciences (CAS), Beijing, 100029 China; 4 Research Institute for Applied Mechanics (RIAM), Kyushu University, Kasuga, Fukuoka 816-8580 Japan

**Keywords:** Environmental chemistry, Environmental impact, Atmospheric chemistry

## Abstract

Changes in the aerosol composition of sulfate (SO_4_^2−^) and nitrate (NO_3_^−^) from 2012 to 2019 have been captured as a paradigm shift in the region downwind of China. Specifically, SO_4_^2−^ dramatically decreased and NO_3_^−^ dramatically increased over downwind locations such as western Japan due to the faster reduction of SO_2_ emissions than NO_*x*_ emissions and the almost constant trend of NH_3_ emissions from China. Emissions from China sharply decreased during COVID-19 lockdowns in February–March 2020, after which China’s economic situation seemed to recover going into 2021. Given this substantial change in Chinese emissions, it is necessary to clarify the impact of long-range PM_2.5_ transport into the leeward of East Asia. In this study, ground-based aerosol compositions observed at three sites in western Japan were analysed. The concentrations of PM_2.5_, SO_4_^2−^ and NO_3_^−^ decreased in 2020 (during COVID-19) compared with 2018–2019 (before COVID-19). In 2021 (after COVID-19), PM_2.5_ and NO_3_^−^ increased and SO_4_^2−^ was unchanged. This suggests the returning long-range PM_2.5_ transport in 2021. From numerical simulations, the status of Chinese emissions during COVID-19 did not explain this returning impact in 2021. This study shows that the status of Chinese emissions in 2021 recovered to that before COVID-19.

## Introduction

Before the outbreak of COVID-19, strong emission regulations in China had led to a rapid decrease in PM_2.5_ concentrations from 2013^1–3^, and this improvement in Chinese PM_2.5_ pollution was associated with improved air quality in terms of PM_2.5_ over the region downwind of China in 2010–2019^4^. Moreover, nitrate aerosol (NO_3_^−^) became an important component of PM_2.5_ by replacing sulfate aerosol (SO_4_^2–^), which had previously dominated aerosol pollution over East Asia^5,6^. This feature is recognised as a paradigm shift in PM_2.5_ composition. Such variations with increasing importance of NO_3_^−^ were also found from long-term analyses of the precipitation chemistry over East Asia^7,8^. These changes were due to the different reduction rates of NO_*x*_ and SO_2_ emissions with the faster reduction in SO_2_ emission and the steadily high rate of NH_3_ emission in China. This trend was expected to continue.

However, unexpected changes in air quality occurred because of the COVID-19 outbreak in December 2019 in the city of Wuhan in Hubei, China^9^. In response to the pandemic, lockdowns were imposed on many cities in China during February and March 2020, and these measures limited human activities such as travel and economic activity^10,11^. Accordingly, strong reductions in anthropogenic emissions were estimated^12^ and observed^13–17^, and subsequent improvements in air pollution were reported over China^18,19^. Because the long-range transport (i.e., trans-boundary transport) of air pollutants in East Asia is an important environmental issue^20,21^, the reduction in anthropogenic emissions in China during COVID-19 will further impact the changes in air pollution over not only China but also downwind regions such as the Korean Peninsula and Japan. This leads to another question: How is the situation after COVID-19? Based on China’s estimated GDP, its economy has recovered since the 2020 lockdowns and is projected to keep growing^22^. Unlike the OECD countries, China achieved positive GDP growth in 2020, and this economic recovery likely led to a rebound in anthropogenic emissions. Against this background, it is worth investigating and clarifying, from the perspective of the downwind region, the changes in long-range transport that resulted from the substantial emission changes that happened before, during and after COVID-19 in China. In the present study, we analysed the changes in aerosol components over the leeward of East Asia based on a combination of ground-based and satellite observations and sensitivity experiments in numerical modeling simulations.

## Results

**Figure 1:**
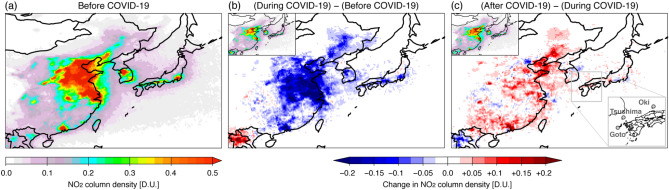
Satellite-measured tropospheric NO_2_ column density over East Asia: (**a**) before COVID-19; (**b**) difference between during COVID-19 and before COVID-19; (**c**) difference between after COVID-19 and during COVID-19. The analysed period is the average of February and March. The inset in the upper left of (**b**) and (**c**) shows the absolute values during and after COVID-19, respectively. The inset in the lower right of (**c**) shows the three observation sites analysed in this study. The maps were generated with gtool3 (http://www.gfd-dennou.org/library/gtool/index.htm.en).

We analysed aerosol compositions for February and March in each year from 2018 to 2021. For simplicity in the following discussion, 2018, for example, stands for the average in February and March in 2018. These two months (February and March) corresponded to COVID-19 lockdown periods in China in 2020^16^ and so are considered as the most suitable period for evaluating the change in PM_2.5_ pollution before, during and after COVID-19. Herein, we refer to the two-year average for 2018 and 2019 as before COVID-19, 2020 as during COVID-19 and 2021 as after COVID-19. Figure 1 shows the NO_2_ column over East Asia as measured by the Ozone Monitoring Instrument (OMI) (see “Methods”) before, during, and after COVID-19. A high NO_2_ column was found over north-eastern China before COVID-19 (Fig. 1a). Compared with its before-COVID-19 status, the NO_2_ column decreased dramatically during COVID-19 over the whole of eastern China (Fig. 1b). Then, although some parts showed decreases, almost all parts of China showed an increased NO_2_ column after COVID-19 compared to during COVID-19 (Fig. 1c). These satellite results suggest substantial deceases in emissions over China in 2020 and then increased emissions along with the economic recovery in 2021.

**Figure 2:**
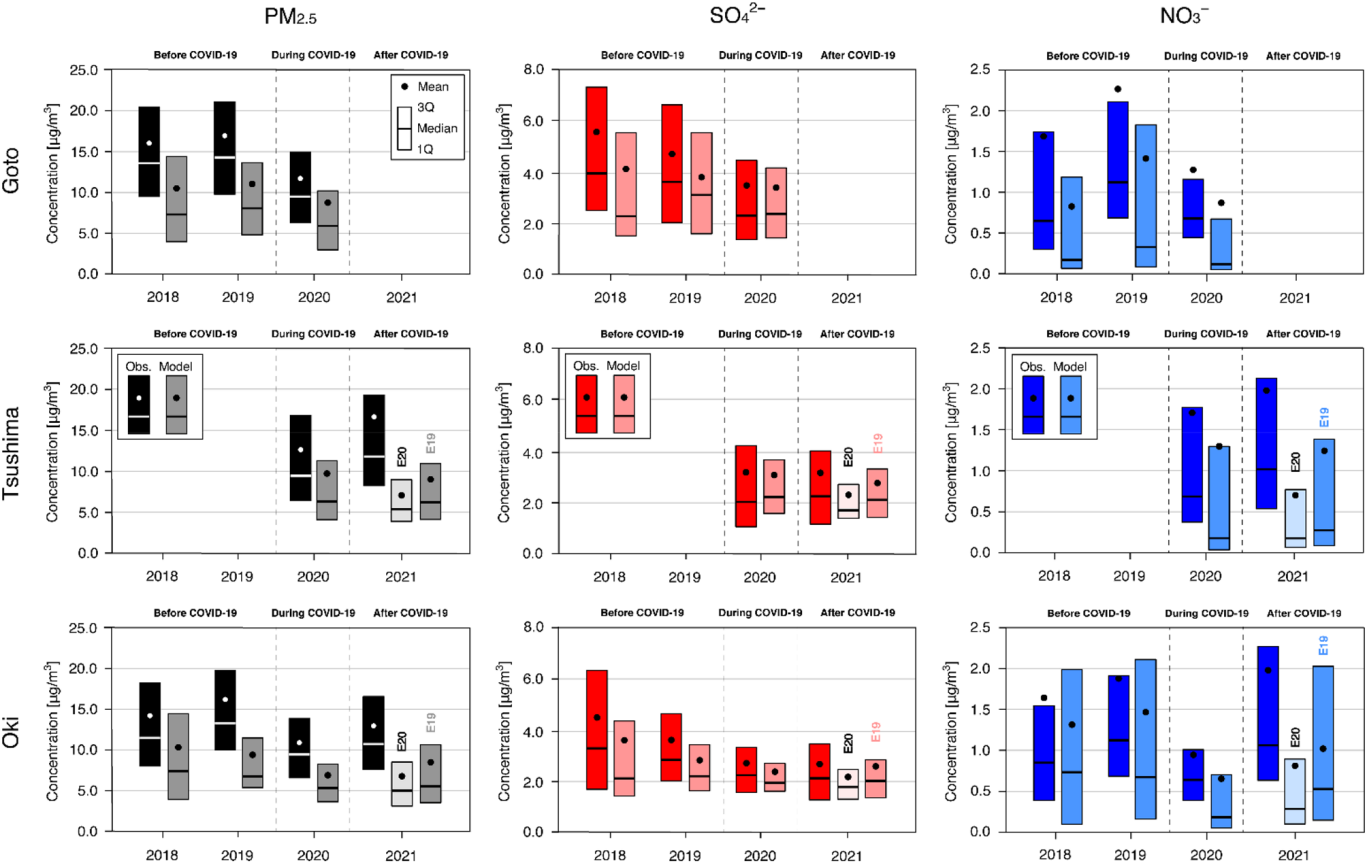
Two-month (February and March) average of (left) PM_2.5_, (centre) SO_4_^2−^ and (right) NO_3_^−^ concentrations from 2018 to 2021 at (top) Goto, (middle) Tsushima and (bottom) Oki. For each year, the observation is shown on the left and the model is shown on the right. For 2021, the model experiments of M21E20 and M21E19 are shown by the lighter colour and darker colour and denoted as E20 and E19, respectively.

**Figure 3:**
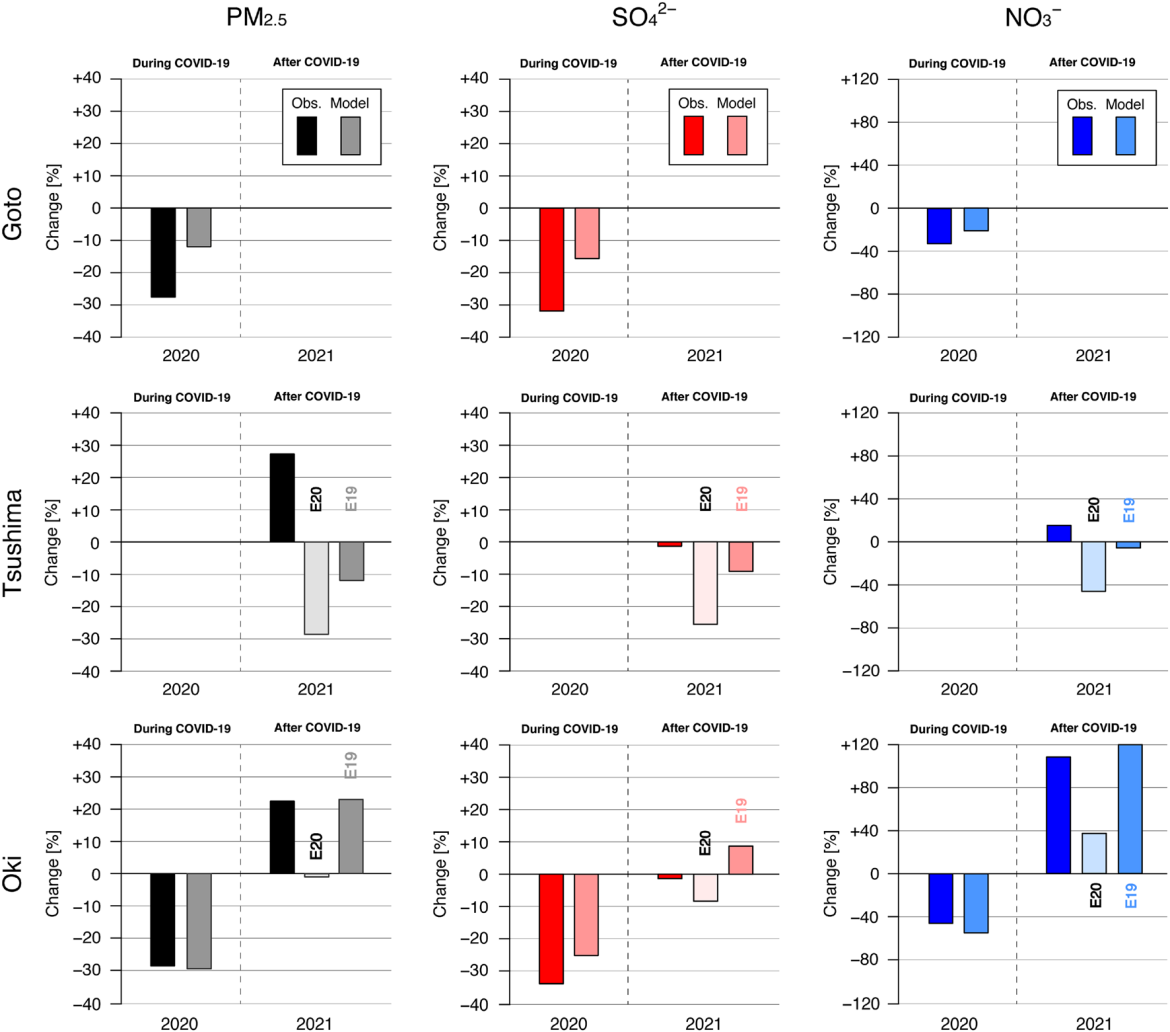
Changes in two-month (February and March) averaged (left) PM_2.5_, (centre) SO_4_^2−^ and (right) NO_3_^−^ concentrations during and after COVID-19 at (top) Goto, (middle) Tsushima and (bottom) Oki. These were calculated from the mean values in Fig. 2. For each year, the observation is shown on the left and the model is shown on the right. For 2021, the model experiments of M21E20 and M21E19 are shown by the lighter colour and darker colour and denoted as E20 and E19, respectively.

Figure 2 shows the observational results of mean, 75 percentile (3Q), median, and 25 percentile (1Q) for changes in PM_2.5_ and its composition at three remote sites in Goto, Tsushima, and Oki located in western Japan (see the lower-right corner of Fig. 1c) from 2018 to 2021. At each of these three sites, an aerosol chemical speciation analyszer (ACSA) measured the PM_2.5_, SO_4_^2−^ and NO_3_^−^ concentrations (see “Methods”). Because the NO_3_^−^ concentration had a high peak in a short time and was otherwise generally close to zero, the mean value is sometimes higher than the 3Q value. In addition to the concentration analyses in Fig. 2, changes in the concentration of PM_2.5_ and its composition (denoted as $C$) during and after COVID-19 are further illustrated in Fig. 3 based on the following equations:

1 $$\text{Change in }{C}_{\text{during COVID-19}}\text{ [\%] =}\frac{{C}_{\text{during COVID-19}} \, - \, {C}_{\text{before COVID-19}}}{{C}_{\text{before COVID-19}}}\times 100,$$

2 $$\text{Change in }{C}_{\text{after COVID-19}}\text{ [\%] =}\frac{{C}_{\text{after COVID-19}} \, - \, {C}_{\text{during COVID-19}}}{{C}_{\text{during COVID-19}}}\times 100.$$

The observational results (Figs. 2 and 3) show that the PM_2.5_, SO_4_^2−^ and NO_3_^−^ concentrations decreased substantially during compared with before COVID-19. The SO_4_^2−^ concentration decreased gradually from 2018 to 2020, whereas the NO_3_^−^ concentration increased from 2018 to 2019 and then decreased from 2019 to 2020. The increase in NO_3_^−^ concentration in 2019 found at Goto and Oki was consistent with our previous study that reported the change in aerosol composition as a paradigm shift^4^. In 2021, increases in PM_2.5_ and NO_3_^−^ and almost the same level of SO_4_^2−^ have been measured. Considering the expected economic recovery suggested from the NO_2_ column (Fig. 1c), the returning impact of long-range transport can be assumed. However, because PM_2.5_ concentration and composition can also be affected by the meteorological field and emission sources other than anthropogenic sources, further discussion is required, and we conducted modeling simulations to clarify this point.

## Discussion

To identify a potential reason for the variation in air pollutants found in 2021, we conducted numerical simulations for 2018–2021. The experiments are described in Table 1 (see “Methods” for details about the modeling configurations). In the names of these experiments, ‘M’ and ‘E’ denote meteorology and Chinese anthropogenic emissions, respectively (e.g., M18E18 indicates the meteorological field for 2018 and the emission inventory for 2018). From 2018 to 2020, the meteorological field and available emissions corresponding to the simulation periods were used. Through comparison with ACSA observations for 2018–2020 (Figs. 2 and 3), we confirmed that the model generally captured the observed variations of PM_2.5_, SO_4_^2−^ and NO_3_^−^ concentrations. Statistical analyses were conducted to evaluate the modeling performance based on the correlation coefficient (*R*), the normalised mean bias (*NMB*) and the normalised mean error (*NME*), and the modeling performance was judged based on metrics from a review of modeling applications (see “Methods” for definitions). The results of the statistical analyses are given in Table 2. For PM_2.5_, the model showed moderate correlation with observations but tended to underestimate the observed concentration; in some cases, the criteria for acceptable model performance were judged as not having been met. This underestimation stemmed mainly from the underestimation of organic aerosol in comparison with a model intercomparison study^23^. For SO_4_^2−^ and NO_3_^−^, the model also tended to underestimate the observed concentrations, but in most cases it was judged to have performed well within the criteria. The model’s underestimation for SO_4_^2−^ was due to the insufficient oxidation process in the current modeling^24^. Considering our previous findings from a model intercomparison study in Japan^25^, one of the possible reasons for the model’s underestimation for NO_3_^−^ was the uncertainty of NH_3_ emissions. In addition to emissions, partitioning between fine- and coarse-mode NO_3_^−^, and also the thermodynamic equilibrium between fine-mode NO_3_^−^ and gas-phase nitric acid (HNO_3_), would be an important aspect to refine the modeling performance on fine-mode NO_3_^−^^26^.

**Table 1:** Description of present modeling experiments (‘M’ and ‘E’ denote meteorology and Chinese anthropogenic emissions, respectively).

Modeling experiment	Meteorology	Anthropogenic emissions from China	Ship emissions	Natural emissions (biomass burning, biogenic and volcanoes)	Purpose
M18E18	2018	2018	2010	2018	Simulation using meteorology and emissions for 2018
M19E19	2019	2019	2010	2019	Simulation using meteorology and emissions for 2019
M20E20	2020	2020	2020	2020	Simulation using meteorology and emissions for 2020
M21E20	2021	2020	2020	2021	Simulation using meteorology and fixed Chinese emissions in 2020 for 2021 assuming the emission levels during COVID-19
M21E19	2021	2019	2020	2021	Simulation using meteorology and fixed Chinese emissions in 2019 for 2021 assuming the emission levels before COVID-19

**Table 2:** Statistical analyses of modeling performance.

Location	Pollutant	Metrics	Modeling experiment				
			M18E18	M19E19	M20E20	M21E20	M21E19
Goto	PM_2.5_	*R*	0.83**	0.81**	0.76**	–	–
		*NMB* (%)	− 37.1	− 34.7	− 22.1*	–	–
		*NME* (%)	40.5*	40.3*	44.4*	–	–
	SO_4_^2−^	*R*	0.79**	0.76**	0.48*	–	–
		*NMB* (%)	− 25.6*	− 17.4*	− 3.2**	–	–
		*NME* (%)	38.4*	37.3*	59.3	–	–
	NO_3_^−^	*R*	0.57	0.60	0.83	–	–
		*NMB* (%)	− 51.2*	− 37.2*	− 32.8*	–	–
		*NME* (%)	70.6*	66.8*	63.7**	–	–
Tsushima	PM_2.5_	*R*	–	–	0.71**	0.59*	0.65*
		*NMB* (%)	–	–	− 22.6*	− 56.6	** − 46.5**
		*NME* (%)	–	–	48.5*	57.8	49.6*
	SO_4_^2−^	*R*	–	–	0.55*	0.66*	0.69*
		*NMB* (%)	–	–	− 2.1**	− 26.1*	− 10.3*
		*NME* (%)	–	–	53.6	48.1*	45.9*
	NO_3_^−^	*R*	–	–	0.81	0.65	**0.65**
		*NMB* (%)	–	–	− 22.9*	− 63.6*	− 36.5*
		*NME* (%)	–	–	58.6**	73.0*	63.8**
Oki	PM_2.5_	*R*	0.75**	0.66*	0.48*	0.61*	0.62*
		*NMB* (%)	− 29.3*	− 42.3	− 37.1	− 49.2	** − 36.8**
		*NME* (%)	39.1*	46.2*	54.0	54.5	47.7*
	SO_4_^2−^	*R*	0.72**	0.71**	0.55*	0.71**	0.69*
		*NMB* (%)	− 20.0*	− 22.7*	− 10.8*	− 17.2*	− 1.8**
		*NME* (%)	42.6*	35.8*	40.8*	38.1*	40.1*
	NO_3_^−^	*R*	0.51	0.54	0.56	0.50	**0.53**
		*NMB* (%)	− 16.9*	− 20.8*	− 32.5*	− 55.3*	− 22.7*
		*NME* (%)	76.4*	70.1*	74.8*	75.2*	74.8*

Notes: for PM_2.5_ and SO_4_^2−^, the model performance goals (marked by **) are *R* > 0.70, *NMB* <  ± 10% and *NME* <  + 35%, and the model performance criteria (marked by *) are *R* > 0.4, *NMB* <  ± 30% and *NME* <  + 50%; for NO_3_^−^, the model performance goals (marked by **) are *NMB* <  ± 15% and *NME* <  + 65%, and the model performance criteria (marked by *) are *NMB* <  ± 65% and *NME* <  + 115%^64^. Where M21E19 is superior to M21E20 in a statistical score, the value is given in bold font.

For 2021, two experiments were conducted because of the lack of emission information for that year (Table 1). In these experiments, anthropogenic emissions from China have been perturbed. One was M21E20, using the meteorological field for 2021 but the Chinese emissions for 2020 (as during COVID-19) with the natural emissions for 2021; the other was M21E19, also using the meteorological field for 2021 but now the Chinese emissions for 2019 (as before COVID-19) with the natural emissions for 2021. Based on these two experiments, the impact of supposing emission changes in 2021 can be estimated. From comparing the simulation results and the ACSA observations for Tsushima and Oki in 2021 (Fig. 2), the observations showed increases in PM_2.5_ and NO_3_^−^ but almost none in SO_4_^2−^, whereas M21E20 showed decreases except for a slight increase in NO_3_^−^ at Oki. The results from M21E20 did not explain the variation observed in 2021, whereas M21E19 gave almost flat variations in PM_2.5_ and SO_4_^2−^ and an increase in NO_3_^−^. The results from M21E19 were much closer to the observational results. The relative changes in the PM_2.5_, SO_4_^2−^ and NO_3_^−^ concentrations after COVID-19 (Fig. 3) also clarified the differences in modeling performance for 2021. Experiment M21E20 showed negative changes after COVID-19 except for NO_3_^−^ at Oki, results that are not consistent with the observations, whereas M21E19 showed better agreement with the observations. These analyses show that M21E19 outperformed M21E20 in capturing the changes observed after COVID-19. In terms of statistical scores (Table 2), M21E19 also outperformed M21E20. These results clarify that the emission status in 2021 was not the same as during COVID-19 and was close to the status before COVID-19. Chinese economic activity and anthropogenic emissions recovered in 2021 and impacted the air quality in downwind regions through long-range PM_2.5_ transport.

**Figure 4:**
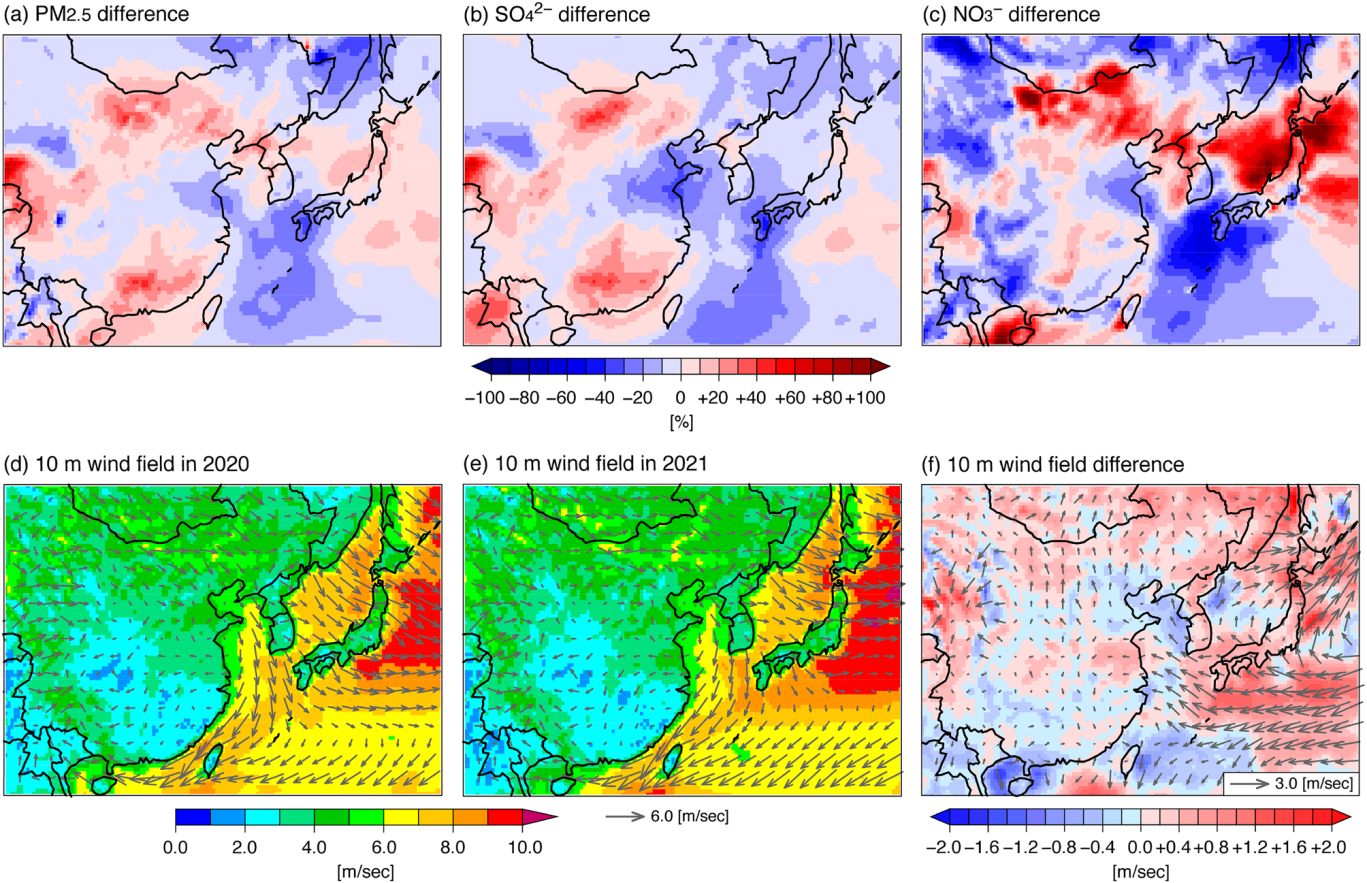
Changes in (**a**) PM_2.5_, (**b**) SO_4_^2−^ and (**c**) NO_3_^−^ concentrations during and after COVID-19 due to meteorology and changes in natural emissions by calculating the M21E20 values minus the M20E20 ones. Modelled 10-m wind field (colour denotes 10-m wind speed) in (**d**) 2020 (M20) and (**e**) 2021 (M21), and change in 10-m wind field by calculating the M21 WRF meteorological field minus the M20 one. The analysed period is the average of February and March. The maps were generated with gtool3 (http://www.gfd-dennou.org/library/gtool/index.htm.en).

Note that experiment M21E20 gave negative changes after COVID-19. Based on the differences between M20E20 and M21E20 (i.e., the M21E20 results minus the M20E20 ones), the impact due to the meteorology and changes in natural emission in 2021 can be investigated. The impacts on air quality caused by the meteorological field have been reported with regard to chemical processes^27,28^ and transport process^29^. The model-estimated changes in the concentrations of PM_2.5_, SO_4_^2−^ and NO_3_^−^ caused by meteorology and natural emissions are shown in Fig. 4a–c. The negative changes in PM_2.5_, SO_4_^2−^ and NO_3_^−^ concentrations over the East China Sea to the Korean Peninsula and western Japan are shown clearly (Fig. 4a–c). Based on the analyses of changes in natural emissions, volcanoes distributed mainly over the western island of Kyushu^30,31^ showed SO_2_ emissions of 605 Gg/period in 2020 and 307 Gg/period in 2021. This decrease could partly explain the negative change in 2021 as found in SO_4_^2−^, but a negative change in SO_4_^2−^ will lead to a positive change in NO_3_^−^ as examined previously as a paradigm shift in aerosol composition. Therefore, the negative changes seen for PM_2.5_, SO_4_^2−^ and NO_3_^−^ can be attributed to changes in the meteorological field. To clarify this point, several meteorological fields are shown in Fig. S1 as the difference between 2021 and 2020 (i.e., the M21 meteorological fields minus the M20 ones) overlaid with the observational results. The positive relative humidity over the East China Sea may enhance the aqueous-phase reaction of SO_4_^2-^ and did not relate to negative changes in PM_2.5_ and SO_4_^2−^ (Fig. S1a). The change in precipitation was inhomogeneous over the East China Sea to western Japan (Fig. S1b). Lower planetary boundary layer (PBL) height found over oceans will lead to accumulated pollutants and did not explain the negative concentrations (Fig. S1c). The change in the 2-m temperature partly explains the NO_3_^−^ change as understood by thermodynamic NO_3_^−^ production. The positive and negative temperatures over western and eastern Japan (Fig. S1d) are generally consistent with the negative and positive changes in NO_3_^−^ concentration over these areas (Figs. 4c and S1d). The positive change in the 10-m wind speed could contribute to the negative changes in concentrations because of the faster transport of polluted air mass (Fig. S1e). The most plausible explanation comes from the change in the 10-m wind direction (Fig. S1f.), which was positive over eastern China to the East China Sea and western Japan. Further detailed illustrations of wind field are shown in Fig. 4d and e. During winter, the seasonal wind pattern is generally in the northwest direction^5^, and a northwest wind direction was simulated over the East China Sea to western Japan in 2020 (Fig. 4d); however, a north wind direction dominated in 2021 over western Japan (Fig. 4e). Therefore, a positive difference in wind direction with positive wind speed was calculated over western Japan (Figs. 4f and S1e and f). The results observed at Tsushima and Oki are also displayed (Fig. S2), and the observations at Tsushima show clearly the change in wind direction from NNW in 2020 to N in 2021. The results of HYSPLIT backward trajectory^32^ analyses from Goto are also presented (Fig. S3). These show the role of long-range transport from eastern China into western Japan during wintertime, and some trajectories were originated from northeastern China or eastern Japan in 2021; indicating the change of transport pattern in 2021 as also shown by simulated meteorological field (Figs. 4d and e and f). These findings suggest that the meteorological conditions in 2021—especially the wind direction—prevented long-range PM_2.5_ transport. Nevertheless, the observations showed increases in PM_2.5_ and NO_3_^−^ and almost no change in SO_4_^2−^ in 2021. This point supports the assertion that the changes in concentrations in 2021 cannot be explained without the returning impact of long-range PM_2.5_ transport associated with the growth in Chinese anthropogenic emissions after COVID-19.

**Figure 5:**
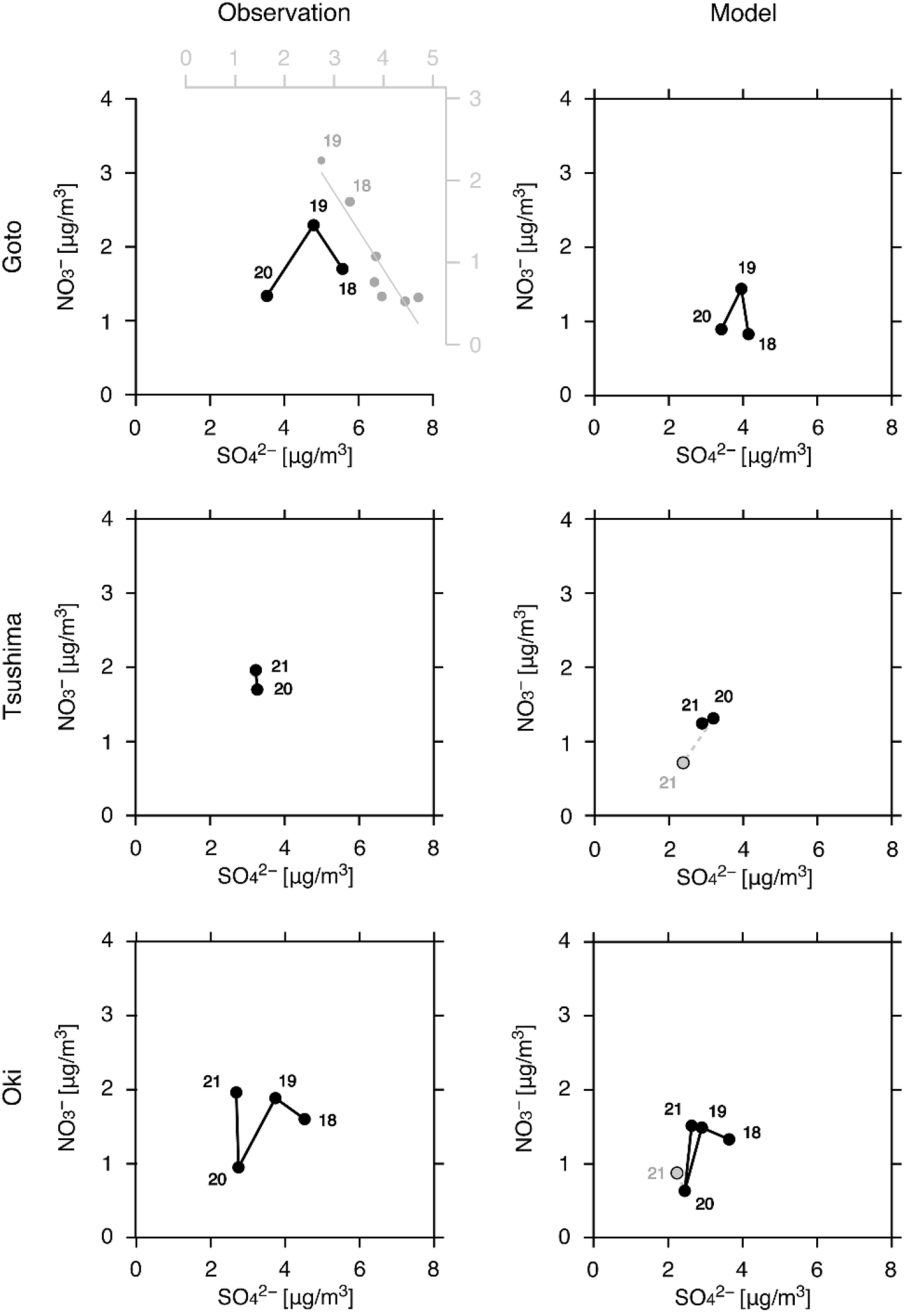
(Left) Observed and (right) modelled scatterplots of SO_4_^2−^ and NO_3_^−^ at (top) Goto, (middle) Tsushima and (bottom) Oki averaged over two months (February and March). The number near each circle indicates the year (i.e., ‘18’ means 2018). At Goto, the results from a previous study^4^ are shown in grey. The previous study analysed PM_1_ components, whereas the present study analysed PM_2.5_ components. For the model results, those of experiment M21E20 are shown by the grey closed circles.

Finally, as an extension of the analysis in our previous study^4^, the SO_4_^2−^ and NO_3_^−^ variations before, during and after COVID-19 are summarised in Fig. 5. The scatterplot of SO_4_^2−^/NO_3_^−^ at Goto from before to during COVID-19 has an inverted V shape. In this figure, the variations of SO_4_^2−^ and NO_3_^−^ reported as a paradigm shift in our previous study^4^ are overlaid (grey open circles with a linear fitting line). These observation data^4^ were measured using a quadruple-type aerosol chemical speciation monitor (Q-ACSM) for fine aerosols of PM_1_, whereas the present study analysed PM_2.5_ components using an ACSA. The variations of SO_4_^2−^ decrease and NO_3_^−^ increase were also found based on ACSA observations up to 2019, and the dramatic decreases in both SO_4_^2−^ and NO_3_^−^ during COVID-19 were furthermore clarified in the present study. The modeling simulation also reproduced well this inverted V-shaped variation. At Tsushima, the variations from during COVID-19 to after COVID-19 were relatively small. From the observational results, SO_4_^2−^ did not change but NO_3_^−^ increased slightly, and the model results also show small variations. Note that experiment M21E20 (grey closed circles) gave deviations from these small variations in both SO_4_^2−^ and NO_3_^−^. These results also show that assuming the emission status from during COVID-19 for 2021 was not appropriate. For further improvement of the modeling for Tsushima, the updated emission status over the Republic of Korea should be used. At Oki, the scatterplot of SO_4_^2−^/NO_3_^−^ from before to during COVID-19 also has an inverted V shape as found at Goto. The variation from during COVID-19 to after COVID-19 shows no change in SO_4_^2−^ but an increase in NO_3_^−^. Experiment M21E19 reproduced this variation, but M21E20 did not. These analyses of the scatterplot of SO_4_^2−^/NO_3_^−^ show substantial decreases in both SO_4_^2−^ and NO_3_^−^ during COVID-19 but suggest the return to the situation of aerosol composition change regarding the importance of NO_3_^−^ after COVID-19 over the leeward of East Asia. The status of aerosol composition change should be monitored continuously because the nitrogen burden over East Asia is an issue of concern both currently and for the future^33,34^. The atmospheric input of nitrogen via dry and wet deposition processes over East Asia has been estimated in previous studies, and its significance has been clarified^35–38^. Targeting the proper management of nitrogen, the nitrogen burden during and after COVID-19 should be clarified in future work.

## Methods

### Observations

To obtain the spatial distribution of the NO_2_ column, which can be regarded as a proxy for NO_*x*_ emissions^39^, space-borne OMI measurements using the level-3 daily global nitrogen dioxide product (OMNO2d version 3.0) gridded into 0.25° × 0.25°^40^ was used. We analysed the tropospheric column with clouds screened under the condition of a cloud fraction of less than 30%.

For ground-based observation of PM_2.5_ and its components, ACSA observation datasets for 10 sites in Japan were available^41^. The Japanese Ministry of the Environment (MOE) created this observation network in April 2017, and two remote observation sites at Goto (128.65°E, 32.60°N) and Oki (133.20°E, 36.28°N), located in western Japan, were analysed in the present study. The ACSA measured PM_2.5_ and the secondary inorganic components of SO_4_^2−^ and NO_3_^−^ with an hourly temporal resolution; this high temporal resolution minimises the effect of the volatilisation of NO_3_^−^^42^. Observations at the Goto site were unavailable from the autumn of 2020 onwards because of a typhoon; to compensate for these missing observations at Goto, observations at the Tsushima (129.29°E, 34.24°N) site were used for 2020 and 2021. The locations of the three sites at Goto, Tsushima and Oki are shown in Fig. 1c.

The lockdown in Wuhan began on 23 January 2020 and lasted until 8 April 2020, and those in other Chinese cities occurred within this period^43^. In Japan, a state of emergency was proclaimed for seven prefectures including Tokyo on 7 April 2020, and this was extended nationwide on 16 April 2020; it ended on 14 May 2020 for 39 prefectures except for mega-cities such Osaka and Tokyo, and on 25 May 2020 for all prefectures in Japan^44^. Emission changes over Japan can be supposed to have been low from February to March. As also seen from the Stringency Index released from One World Data^45^, the countermeasures against COVID-19 were the strictest in China from late January to the end of March, whereas other countries in East Asia had relatively loose measures in place during this period. Given these differences between China and Japan, the period analysed in the present study was chosen as February to March to focus on the variation of long-range PM_2.5_ transport according to Chinese anthropogenic emissions.

### Numerical modeling simulations

The numerical modeling simulations were performed using the regional chemical transport model of the Comprehensive Air quality Model with eXtensions (CAMx) version 6.4^46^. The simulation domain covering East Asia was configured by 215 × 120 grid points with 36-km horizontal resolution, centred at 116.2°E and 37.8°N on a Lambert conformal projection. The vertical resolution was 44 non-uniformly spaced layers from the surface to 50 hPa^37^. The meteorological fields to drive CAMx were calculated by the Weather Research and Forecasting (WRF) model version 4.1.1^47^. Gas-phase and aerosol chemistry were respectively adopted for SAPRC07TC^48,49^ and the CF scheme in CAMx. Based on previous findings^50,51^, the NO_2_ aqueous-phase reaction in the SO_4_^2−^ oxidation process was added. In addition, to enhance the production of SO_4_^2−^ during winter haze events, the heterogeneous reaction of SO_2_ on aerosol surfaces was introduced following the work on Chinese haze events^52^. The initial and lateral boundary conditions were prepared from winter-averaged values of the extended CMAQ model over the northern hemisphere^53^.

In the present study, five modeling experiments were conducted, and the simulation setups are described in Table 1. In the names of these experiments, ‘M’ and ‘E’ stand for meteorology and anthropogenic emissions, respectively. For each year, the corresponding meteorological field generated by WRF was used. The emissions inventories were prepared as follows. Anthropogenic emissions except those in Japan and China were based on Hemispheric Transport of Air Pollution (HTAP) version 2.2^54^. For Japan, the latest available domestic emission inventory for 2015 developed by the MOE^55^ was used. For China, the emission projections were based on the Multi-resolution Emission Inventory in China (MEIC). Trends in Chinese emissions up to 2017 have been reported^56^, and their variation during the COVID-19 pandemic in 2020 have also been described^12^. Neither of those two references covered 2018, so instead the average data for 2017 and 2019 were calculated. From the MEIC estimations, the reduction in NO_*x*_ emissions in 2020 compared to those in 2019 during February and March was 36% and 14%, respectively, and the reduction in SO_2_ emissions was 27% and 11%, respectively. NH_3_ emissions were not provided for COVID-19 ^12^, but because their main source is agriculture, we assumed that NH_3_ emissions were unchanged during COVID-19^57^ and we fixed them to the levels for 2017. Biogenic emissions were prepared from the Model of Emissions of Gases and Aerosols from Nature (MEGAN)^58^ using the WRF-simulated meteorological field for the corresponding period. Biomass-burning emissions were taken from the beta version of the Global Fire Emissions Database (GFED) version 4.1^59^. SO_2_ emissions from 15 volcanoes in Japan were prepared from a measurement report by the Japan Meteorological Agency (JMA)^60^. Sulphur emissions from ships could influence air quality, especially in areas close to the sea^61^. Because of the limited ship emission inventory, HTAP version 2.2 was used for 2018 and 2019, and a reduction in SO_2_ emissions of 77% for 2020 and 2021 was applied based on a report by the International Maritime Organization (IMO)^62^. This reduction rate was consistent with a high-resolution emission inventory developed for Japan^63^. To our knowledge, the emission situation in 2021—regarded as after the COVID-19 pandemic—is not yet available, so to consider the emission status in the region downwind of China from the observational facts, two experiments were conducted: (i) M21E20, which assumed the emission levels during COVID-19 for those in 2021, and (ii) M21E19, which assumed the emission levels before COVID-19 for those in 2021. By comparing these two experiments, the emission status in 2021 was investigated.

The model performance was evaluated statistically using the metrics of *R*, *NMB* and *NME* defined as

3 $$R =\frac{{\sum }_{1}^{N}\left({O}_{i}-\overline{O }\right)\left({M}_{i}-\overline{M }\right)}{\sqrt{{\sum }_{1}^{N}{\left({O}_{i}-\overline{O }\right)}^{2}}\sqrt{{\sum }_{1}^{N}{\left({M}_{i}-\overline{M }\right)}^{2}}},$$

4 $$NMB =\frac{\sum_{1}^{N}\left({M}_{i}-{O}_{i}\right)}{{\sum }_{1}^{N}{O}_{i}},$$

5 $$NME =\frac{\sum_{1}^{N}|{M}_{i}-{O}_{i}|}{{\sum }_{1}^{N}{O}_{i}},$$

where *N* is the total number of paired observations (*O*) and models (*M*), and these averages are denoted as $\overline{O }$ and $\overline{M }$, respectively. The recommended metrics based on a literature review for the United States^64^ are given in Table 2.

## Supplementary Information

Supplementary Information.

## Data Availability

The datasets generated during and/or analysed during the current study are available from the corresponding author on reasonable request.
