# Supplementary material for: Returning long-range PM2.5 transport into the leeward of East Asia in 2021 after Chinese economic recovery from the COVID-19 pandemic
Source: Sci Rep. 2022 Apr 1;12:5539. doi: 10.1038/s41598-022-09388-2 (PMC8972671; doi:10.1038/s41598-022-09388-2)
Supplement: Supplementary file 1 — Supplementary Information. [file 41598_2022_9388_MOESM1_ESM.pdf]

Supplementary Information:

Returning long-range $PM_{2.5}$ transport into the leeward of East Asia in 2021 after Chinese economic recovery from the COVID-19 pandemic

Syuichi Itahashi[1,*], **Yuki Yamamura**[2], **Zhe Wang**[3], **and Itsushi Uno**[4]

[1] Sustainable System Research Laboratory (SSRL), Central Research Institute of Electric Power Industry (CRIEPI), Abiko, Chiba 270-1194, Japan

[2] Fukuoka Institute of Health and Environmental Science, Dazaifu, Fukuoka 818-0135, Japan

[3] State Key Laboratory of Atmospheric Boundary Layer Physics and Atmospheric Chemistry (LAPC), Institute of Atmospheric Physics (IAP), Chinese Academy of Sciences (CAS), Beijing 100029, China

[4] Research Institute for Applied Mechanics (RIAM), Kyushu University, Kasuga, Fukuoka 816-8580, Japan

[*]Corresponding author: isyuichi@criepi.denken.or.jp

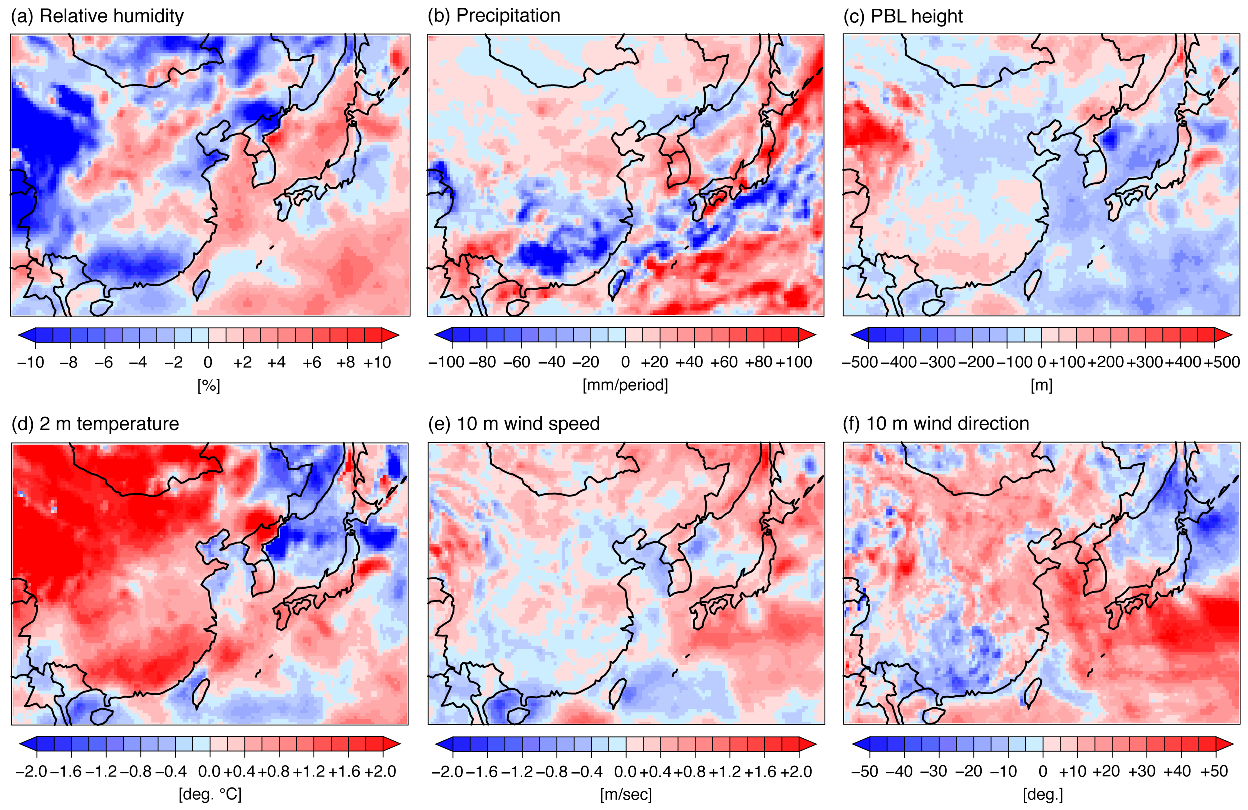

Supplementary Figure S1. Changes in the meteorological parameters of (a) relative humidity, (b) precipitation, (c) planetary boundary layer (PBL) height, (d) 2-m temperature, (e) 10-m wind speed and (f) 10-m wind direction by calculating the M21 WRF meteorological field minus the M20 one. The analysed period is the average of February and March. The observational results at Tsushima and Oki are overlaid with circles. The maps were generated with gtool3 (http://www.gfd-dennou.org/library/gtool/index.htm.en).

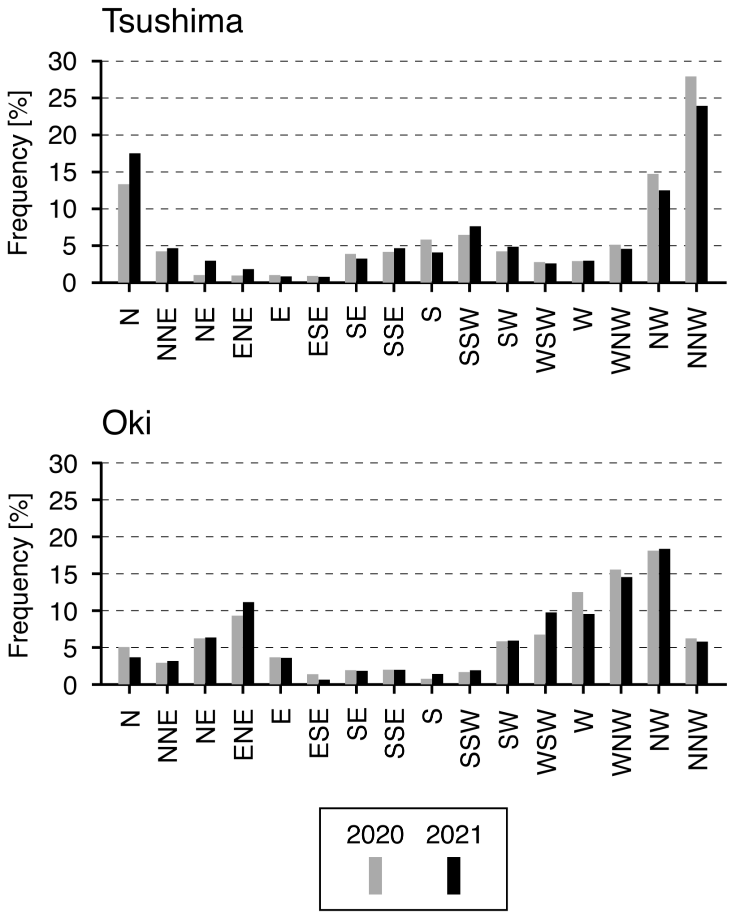

Supplementary Figure S2. Observed surface wind direction in 16 directions at Tsushima and Oki analysed over two months (February and March).

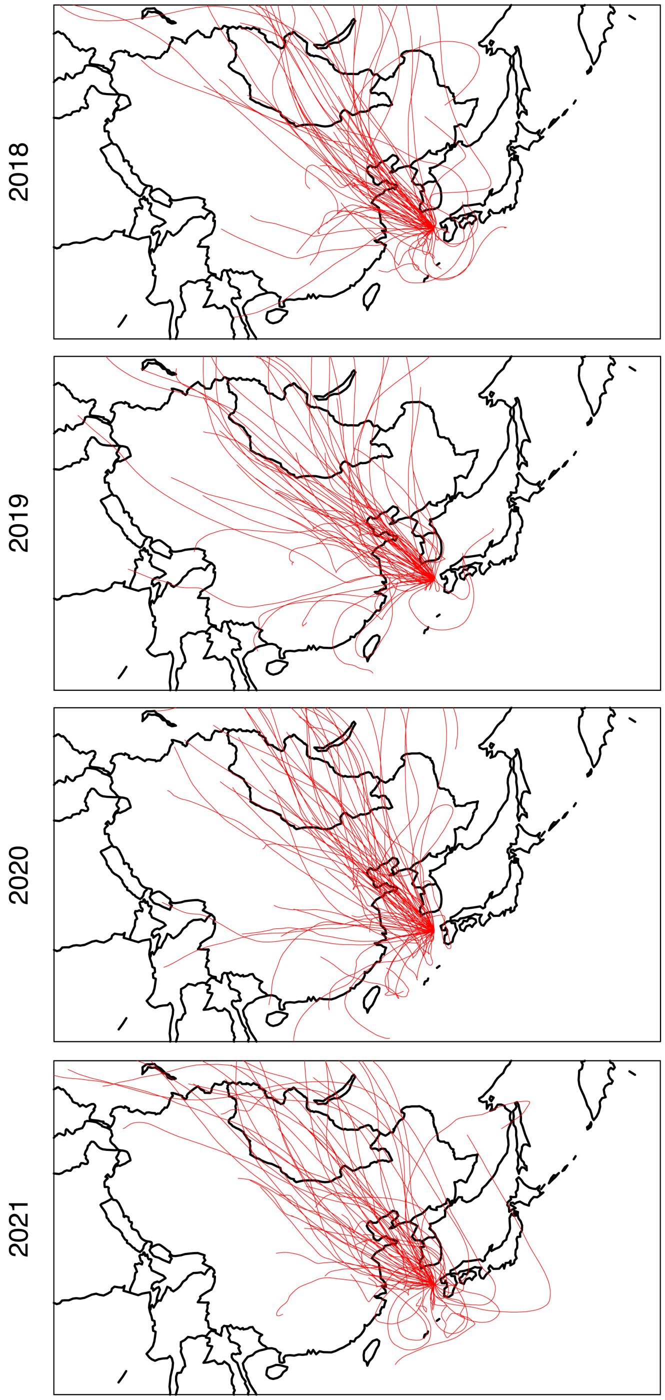

Supplementary Figure S3. HYSPLIT backward-trajectory results started from Goto (128.65°E, 32.60°N) at 2000 m above ground level at 1200 local time on each day during February and March. The trajectories were mapped into the modeling domain used in this study. The maps were generated with gtool3 (http://www.gfd-dennou.org/library/gtool/index.htm.en).
